# 5,17-Dibromo-26,28-bis­[(meth­oxy­carbon­yl)meth­oxy]-25,27-diprop­oxy-2,8,14,20-tetra­thia­calix[4]arene

**DOI:** 10.1107/S1600536812014559

**Published:** 2012-04-13

**Authors:** Li-Jing Zhang, Ling-Ling Liu, Qi-Kui Liu, Dian-Shun Guo

**Affiliations:** a Department of Chemistry, Shandong Normal University, Jinan 250014, People’s Republic of China

## Abstract

The title thia­calix[4]arene derivative, C_36_H_34_Br_2_O_8_S_4_, adopts an unusual pinched cone conformation with the prop­oxy-substituted benzene rings inclined inward [forming a dihedral angle of 33.4 (1)°] and with the brominated benzene rings bent outward, making a dihedral angle of 66.1 (1)°. In the crystal, the mol­ecules form chains along [001] *via* C—H⋯S hydrogen bonds and S⋯S contacts [S⋯S = 3.492 (3) Å]. The chains are associated into bilayers through C—H⋯O hydrogen bonds, generating an *R*_2_^2^(10) motif.

## Related literature

For general background to the chemistry of thia­calix[4]arenes, see: Shokova & Kovalev (2003 ▶); Lhoták (2004 ▶); Morohashi *et al.* (2006 ▶); Kajiwara *et al.* (2007 ▶); Guo *et al.* (2007 ▶). For related structures, see: Lhoták *et al.* (2000 ▶, 2003 ▶); Himl *et al.* (2005 ▶); Xu *et al.* (2008 ▶); Chen *et al.* (2010 ▶); Liu *et al.* (2011 ▶). For hydrogen-bond motifs, see: Bernstein *et al.* (1995 ▶); Hu *et al.* (2009 ▶). For atomic van der Waals radii, see: Bondi (1964 ▶).
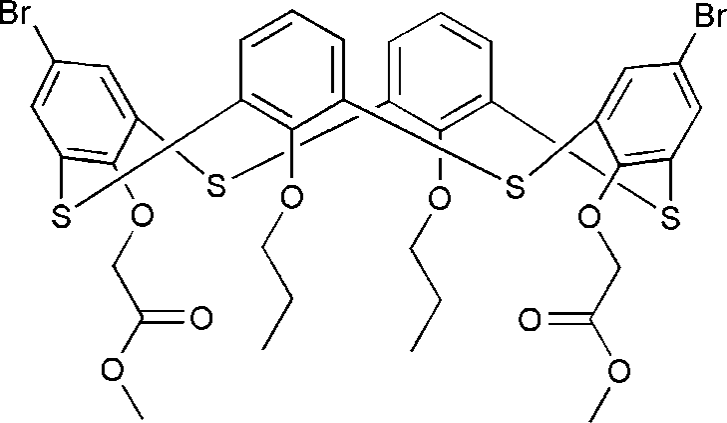


## Experimental

### 

#### Crystal data

C_36_H_34_Br_2_O_8_S_4_
*M* *_r_* = 882.69
Monoclinic, 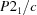

*a* = 16.024 (3) Å
*b* = 14.808 (3) Å
*c* = 15.872 (3) Å
β = 100.065 (3)°
*V* = 3708.3 (12) Å^3^
*Z* = 4
Mo *K*α radiation
μ = 2.46 mm^−1^
*T* = 173 K
0.38 × 0.18 × 0.13 mm

#### Data collection

Bruker SMART CCD area-detector diffractometer
Absorption correction: multi-scan (*SADABS*; Bruker, 1999 ▶) *T*_min_ = 0.455, *T*_max_ = 0.740
19322 measured reflections
6989 independent reflections
4908 reflections with *I* > 2σ(*I*)
*R*_int_ = 0.060

#### Refinement

*R*[*F* ^2^ > 2σ(*F* ^2^)] = 0.042
*wR*(*F* ^2^) = 0.091
*S* = 0.93
6989 reflections
455 parameters
H-atom parameters constrained
Δρ_max_ = 0.62 e Å^−3^
Δρ_min_ = −0.55 e Å^−3^

### 

Data collection: *SMART* (Bruker, 1999 ▶); cell refinement: *SAINT* (Bruker, 1999 ▶); data reduction: *SAINT*; program(s) used to solve structure: *SHELXS97* (Sheldrick, 2008 ▶); program(s) used to refine structure: *SHELXL97* (Sheldrick, 2008 ▶); molecular graphics: *SHELXTL* (Sheldrick, 2008 ▶); software used to prepare material for publication: *SHELXTL*.

## Supplementary Material

Crystal structure: contains datablock(s) I, global. DOI: 10.1107/S1600536812014559/ld2052sup1.cif

Structure factors: contains datablock(s) I. DOI: 10.1107/S1600536812014559/ld2052Isup2.hkl

Additional supplementary materials:  crystallographic information; 3D view; checkCIF report

## Figures and Tables

**Table 1:** Hydrogen-bond geometry (Å, °)

*D*—H⋯*A*	*D*—H	H⋯*A*	*D*⋯*A*	*D*—H⋯*A*
C4—H4⋯S3^i^	0.95	2.91	3.755 (3)	149
C33—H33*A*⋯O7^ii^	0.98	2.58	3.551 (5)	169

Symmetry codes: (i) 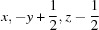; (ii) 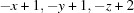.

**Fig. 1.**
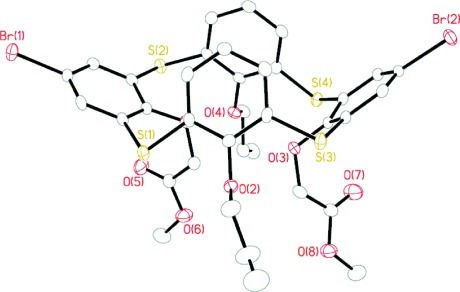
The molecular structure of the title compound with displacement ellipsoids drawn at the 30% probability level for non-H atoms. The hydrogen atoms have been omitted for clarity.

**Fig. 2.**
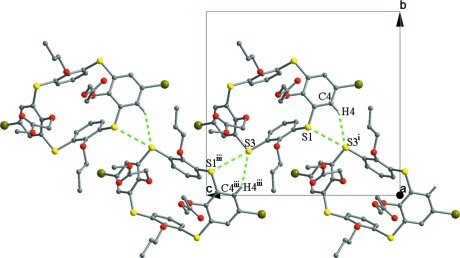
The hydrogen-bonded one-dimensional chain of the title compound, viewed along the crystallographic a axis, showing the C(8) motif. For the sake of clarity, H atoms not involved in the motifs have been omitted. [Symmetry codes: (i) x, -y + 1/2, z - 1/2; (iii) x, -y + 1/2, z + 1/2.]

## supplementary crystallographic information

### Comment

Thiacalix[4]arenes have attracted much interest because of their specific affinity, selectivity in molecular recognition, and supramolecular assembly (Shokova & Kovalev, 2003; Lhoták, 2004; Morohashi *et al.*, 2006; Kajiwara *et al.*, 2007; Guo *et al.*, 2007). Numerous crystal structures of thiacalix[4]arenes uniformly substituted at the lower rim or at the upper rim are known (Lhoták *et al.*, 2000, 2003; Himl *et al.*, 2005). Recently, we have presented structures of several dibromo and tetrabromothiacalix[4]arene derivatives that possess four identical or four different substituents at the lower rim (Xu *et al.*, 2008; Chen *et al.*, 2010; Liu *et al.*, 2011). Here we report the crystal structure of another dibromothiacalix[4]arene compound with different substituents at the lower rim – 5,17-dibromo-26,28-bis[(methoxycarbonyl)methoxy]-25,27-dipropoxy-2,8,14,20-tetrathiacalix[4]arene.

In the crystal structure of the title compound, C_36_H_34_Br_2_O_8_S_4_, (Fig. 1), the thiacalix[4]arene unit adopts an unusual pinched cone conformation. Two opposite bromosubstituted phenolic rings are strongly bent outwards the thiacalix cavity with a Br···Br distance of 13.819 (3) Å [larger than that reported previously - 13.165 (2) Å (Liu *et al.*, 2011)]. The other two opposing phenolic rings are bent inwards. The phenolic rings form dihedral angles of 26.09 (7), 65.57 (7), 40.29 (6) and 81.18 (7)° with the virtual plane defined by the four bridging S atoms.

In the packing, there are several intermolecular short contacts (Table 1). The molecules are linked into an infinite zigzag one-dimensional chain along [001] (Hu *et al.*, 2009) by intermolecular C4—H4···S3(*x*, -*y* + 1/2, *z* - 1/2) hydrogen bonds, locally forming a *C*(8) motif (Bernstein *et al.*, 1995) (Fig. 2). Interestingly, in such a chain all 'tails' of the molecules extend to the same orientation and a significant S···S interaction between the adjacent thiacalix[4]arenes stabilizes the motif, with a S1···S3 distance of 3.492 (3) Å (S = 1.80 Å; Bondi, 1964). Finally, these chains are packed into a complex tail-to-tail-oriented bilayer system by a combination of interchain C—H···O hydrogen bonds, giving an *R*_2_^2^(10) motif.

### Experimental

BrCH_2_CO_2_Me (0.08 ml, 0.84 mmol) was added to a suspension of 5,17-dibromo-26,28-dihydroxy-25,27-dipropoxy-2,8,14,20-tetrathiacalix[4]arene (0.080 g, 0.14 mmol) and anhydrous K_2_CO_3_ (0.022 g, 0.17 mmol) in dry acetone (15 ml). The resulting mixture was stirred for 3 h at 343 K and cooled to room temperature. The solvent was removed under reduced pressure. The residue was neutralized with 5% aqueous HCl and extracted with CH_2_Cl_2_. The organic layer was separated and washed with saturated sodium hydrogen carbonate and brine, and dried over anhydrous MgSO_4_. The solvent was evaporated *in vacuo* and the residue was chromatographed on a silica gel column (CH_2_Cl_2_/petroleum ether = 1:1) to give the title compound as a white solid (yield 63%, *R*_f_ = 0.3) and another product (yield 28%, *R*_f_ = 0.45). ^1^H NMR (300 MHz, CDCl_3_) for the title product: *δ* 7.67 (*s*, 4H), 6.55 (*d*, 4H, *J* = 7.26 Hz), 6.46 (*t*, 2H, *J* = 7.57 Hz), 5.10 (*s*, 4H), 3.88 (*t*, 4H, *J* = 7.47 Hz), 3.74 (*s*, 6H), 1.89~1.77 (*m*, 4H), 1.05 (*t*, 6H, *J* = 7.39 Hz). IR (KBr pellets, cm^-1^): 1763 (C=O). Single crystals suitable for X-ray diffraction analysis were obtained by slow evaporation of a solution in CH_3_OH and CHCl_3_ at 298 K.

### Refinement

All non-hydrogen atoms were refined with anisotropic displacement parameters. Hydrogen atoms attached to carbon atoms were placed in geometrically idealized positions and refined as riding atoms with C—H = 0.98 Å and *U*_iso_(H) = 1.5 *U*_eq_(C) (methyl); C—H = 0.99 Å and *U*_iso_(H) = 1.2 *U*_eq_(C) (methylene); C—H = 0.95 Å and *U*_iso_(H) = 1.2 *U*_eq_(C) (aromatic). The positions of methyl hydrogens were rotationally optimized.

### Crystal data

**Table:**

C_36_H_34_Br_2_O_8_S_4_	*F*(000) = 1792
*M**_r_* = 882.69	*D*_x_ = 1.581 Mg m^−^^3^
Monoclinic, *P*2_1_/*c*	Mo *K*α radiation, λ = 0.71073 Å
*a* = 16.024 (3) Å	Cell parameters from 4762 reflections
*b* = 14.808 (3) Å	θ = 2.4–24.5°
*c* = 15.872 (3) Å	µ = 2.46 mm^−^^1^
β = 100.065 (3)°	*T* = 173 K
*V* = 3708.3 (12) Å^3^	Block, colourless
*Z* = 4	0.38 × 0.18 × 0.13 mm

### Data collection

**Table:**

Bruker SMART CCD area-detector diffractometer	6989 independent reflections
Radiation source: fine-focus sealed tube	4908 reflections with *I* > 2σ(*I*)
Graphite monochromator	*R*_int_ = 0.060
phi and ω scans	θ_max_ = 25.6°, θ_min_ = 1.9°
Absorption correction: multi-scan (*SADABS*; Bruker, 1999)	*h* = −19→10
*T*_min_ = 0.455, *T*_max_ = 0.740	*k* = −18→17
19322 measured reflections	*l* = −17→19

### Refinement

**Table:**

Refinement on *F*^2^	Primary atom site location: structure-invariant direct methods
Least-squares matrix: full	Secondary atom site location: difference Fourier map
*R*[*F*^2^ > 2σ(*F*^2^)] = 0.042	Hydrogen site location: inferred from neighbouring sites
*wR*(*F*^2^) = 0.091	H-atom parameters constrained
*S* = 0.93	*w* = 1/[σ^2^(*F*_o_^2^) + (0.0393*P*)^2^] where *P* = (*F*_o_^2^ + 2*F*_c_^2^)/3
6989 reflections	(Δ/σ)_max_ = 0.001
455 parameters	Δρ_max_ = 0.62 e Å^−^^3^
0 restraints	Δρ_min_ = −0.55 e Å^−^^3^

### Special details

**Table:**

Geometry. All e.s.d.'s (except the e.s.d. in the dihedral angle between two l.s. planes) are estimated using the full covariance matrix. The cell e.s.d.'s are taken into account individually in the estimation of e.s.d.'s in distances, angles and torsion angles; correlations between e.s.d.'s in cell parameters are only used when they are defined by crystal symmetry. An approximate (isotropic) treatment of cell e.s.d.'s is used for estimating e.s.d.'s involving l.s. planes.
Refinement. Refinement of *F*^2^ against ALL reflections. The weighted *R*-factor *wR* and goodness of fit *S* are based on *F*^2^, conventional *R*-factors *R* are based on *F*, with *F* set to zero for negative *F*^2^. The threshold expression of *F*^2^ > σ(*F*^2^) is used only for calculating *R*-factors(gt) *etc*. and is not relevant to the choice of reflections for refinement. *R*-factors based on *F*^2^ are statistically about twice as large as those based on *F*, and *R*- factors based on ALL data will be even larger.

### Fractional atomic coordinates and isotropic or equivalent isotropic displacement parameters (Å^2^)

**Table:**

	*x*	*y*	*z*	*U*_iso_*/*U*_eq_	
Br1	0.11682 (2)	0.60398 (2)	0.21293 (2)	0.04232 (12)	
Br2	0.03760 (2)	0.38070 (3)	1.04064 (3)	0.04869 (13)	
C1	0.20213 (19)	0.6405 (2)	0.4739 (2)	0.0258 (7)	
C2	0.16788 (18)	0.6550 (2)	0.38842 (19)	0.0271 (7)	
H2	0.1460	0.7128	0.3702	0.033*	
C3	0.16546 (19)	0.5857 (2)	0.32980 (19)	0.0274 (7)	
C4	0.19244 (19)	0.5003 (2)	0.35546 (19)	0.0285 (7)	
H4	0.1884	0.4527	0.3148	0.034*	
C5	0.22583 (19)	0.48368 (19)	0.44142 (19)	0.0255 (7)	
C6	0.23380 (18)	0.5547 (2)	0.49994 (19)	0.0256 (7)	
C7	0.17985 (19)	0.3580 (2)	0.54785 (18)	0.0235 (7)	
C8	0.1014 (2)	0.4000 (2)	0.5355 (2)	0.0301 (8)	
H8	0.0837	0.4348	0.4852	0.036*	
C9	0.0489 (2)	0.3920 (2)	0.5948 (2)	0.0324 (8)	
H9	−0.0048	0.4209	0.5852	0.039*	
C10	0.0742 (2)	0.3419 (2)	0.6690 (2)	0.0310 (8)	
H10	0.0381	0.3367	0.7104	0.037*	
C11	0.1524 (2)	0.2996 (2)	0.68202 (19)	0.0271 (7)	
C12	0.20550 (19)	0.30606 (19)	0.62144 (19)	0.0242 (7)	
C13	0.1789 (2)	0.3319 (2)	0.85101 (19)	0.0272 (7)	
C14	0.12273 (19)	0.3240 (2)	0.90827 (19)	0.0298 (8)	
H14	0.0927	0.2692	0.9118	0.036*	
C15	0.1109 (2)	0.3961 (2)	0.9597 (2)	0.0295 (8)	
C16	0.1492 (2)	0.4789 (2)	0.95107 (19)	0.0306 (8)	
H16	0.1377	0.5292	0.9844	0.037*	
C17	0.20401 (19)	0.4872 (2)	0.89340 (19)	0.0267 (7)	
C18	0.22386 (19)	0.4120 (2)	0.84720 (19)	0.0246 (7)	
C19	0.1805 (2)	0.62323 (19)	0.77646 (19)	0.0270 (7)	
C20	0.0935 (2)	0.6079 (2)	0.7646 (2)	0.0320 (8)	
H20	0.0688	0.5829	0.8096	0.038*	
C21	0.0431 (2)	0.6290 (2)	0.6875 (2)	0.0347 (8)	
H21	−0.0162	0.6181	0.6795	0.042*	
C22	0.0782 (2)	0.6657 (2)	0.6223 (2)	0.0315 (8)	
H22	0.0430	0.6797	0.5693	0.038*	
C23	0.1646 (2)	0.68234 (19)	0.63296 (19)	0.0264 (7)	
C24	0.21621 (19)	0.6616 (2)	0.7109 (2)	0.0249 (7)	
C25	0.3470 (2)	0.5133 (2)	0.6152 (2)	0.0320 (8)	
H25A	0.3524	0.4502	0.5965	0.038*	
H25B	0.3578	0.5140	0.6786	0.038*	
C26	0.4131 (2)	0.5699 (2)	0.5838 (2)	0.0358 (8)	
C27	0.5595 (2)	0.5920 (3)	0.5987 (3)	0.0608 (12)	
H27A	0.5548	0.5831	0.5369	0.091*	
H27B	0.6126	0.5655	0.6283	0.091*	
H27C	0.5588	0.6568	0.6113	0.091*	
C28	0.2829 (2)	0.1705 (2)	0.6167 (2)	0.0519 (11)	
H28A	0.2342	0.1425	0.6375	0.062*	
H28B	0.2753	0.1611	0.5541	0.062*	
C29	0.3602 (3)	0.1274 (3)	0.6572 (3)	0.0784 (16)	
H29A	0.3724	0.1453	0.7182	0.094*	
H29B	0.4075	0.1491	0.6300	0.094*	
C30	0.3564 (3)	0.0260 (3)	0.6516 (3)	0.0887 (17)	
H30A	0.3166	0.0032	0.6869	0.133*	
H30B	0.4129	0.0009	0.6722	0.133*	
H30C	0.3373	0.0079	0.5919	0.133*	
C31	0.3558 (2)	0.3663 (2)	0.8103 (2)	0.0387 (9)	
H31A	0.3885	0.3744	0.7634	0.046*	
H31B	0.3375	0.3024	0.8097	0.046*	
C32	0.4124 (2)	0.3854 (2)	0.8935 (2)	0.0351 (8)	
C33	0.5475 (2)	0.3478 (3)	0.9742 (2)	0.0577 (11)	
H33A	0.5603	0.4115	0.9871	0.087*	
H33B	0.5993	0.3163	0.9663	0.087*	
H33C	0.5250	0.3202	1.0217	0.087*	
C34	0.3351 (2)	0.7543 (2)	0.7622 (2)	0.0371 (9)	
H34A	0.3119	0.7636	0.8155	0.045*	
H34B	0.3190	0.8067	0.7241	0.045*	
C35	0.4294 (2)	0.7450 (2)	0.7823 (2)	0.0397 (9)	
H35A	0.4516	0.7370	0.7284	0.048*	
H35B	0.4443	0.6906	0.8179	0.048*	
C36	0.4710 (2)	0.8276 (3)	0.8296 (2)	0.0508 (10)	
H36A	0.4612	0.8806	0.7922	0.076*	
H36B	0.5321	0.8171	0.8459	0.076*	
H36C	0.4465	0.8381	0.8812	0.076*	
O1	0.26345 (13)	0.54324 (13)	0.58541 (13)	0.0284 (5)	
O2	0.28416 (13)	0.26639 (13)	0.63464 (13)	0.0300 (5)	
O3	0.28207 (13)	0.42264 (14)	0.79386 (13)	0.0307 (5)	
O4	0.30214 (13)	0.67228 (13)	0.72072 (13)	0.0302 (5)	
O5	0.40072 (16)	0.62414 (17)	0.52780 (17)	0.0546 (7)	
O6	0.48904 (15)	0.54881 (17)	0.62764 (17)	0.0510 (7)	
O7	0.39604 (17)	0.4311 (2)	0.94997 (18)	0.0649 (8)	
O8	0.48456 (15)	0.34097 (18)	0.89600 (15)	0.0484 (7)	
S1	0.25109 (5)	0.37085 (5)	0.47437 (5)	0.0301 (2)	
S2	0.20796 (5)	0.73180 (5)	0.54737 (5)	0.0321 (2)	
S3	0.18647 (6)	0.24030 (5)	0.77987 (5)	0.0335 (2)	
S4	0.24789 (5)	0.59458 (6)	0.87516 (5)	0.0325 (2)	

### Atomic displacement parameters (Å^2^)

**Table:**

	*U*^11^	*U*^22^	*U*^33^	*U*^12^	*U*^13^	*U*^23^
Br1	0.0565 (3)	0.0436 (2)	0.02588 (19)	0.00021 (18)	0.00459 (16)	0.00639 (16)
Br2	0.0532 (3)	0.0523 (3)	0.0492 (2)	0.01553 (19)	0.03265 (19)	0.0158 (2)
C1	0.0273 (19)	0.0213 (16)	0.0312 (18)	−0.0038 (14)	0.0115 (14)	0.0021 (15)
C2	0.0270 (19)	0.0239 (17)	0.0325 (18)	−0.0030 (14)	0.0110 (14)	0.0077 (15)
C3	0.0261 (19)	0.0338 (19)	0.0229 (17)	−0.0051 (15)	0.0063 (13)	0.0046 (15)
C4	0.039 (2)	0.0250 (17)	0.0244 (17)	−0.0048 (15)	0.0142 (14)	−0.0011 (15)
C5	0.0321 (19)	0.0210 (16)	0.0260 (17)	−0.0008 (14)	0.0121 (14)	0.0032 (14)
C6	0.0243 (18)	0.0281 (18)	0.0257 (17)	−0.0035 (14)	0.0082 (13)	0.0027 (15)
C7	0.0315 (19)	0.0198 (16)	0.0197 (16)	−0.0062 (14)	0.0055 (13)	−0.0032 (13)
C8	0.035 (2)	0.0293 (18)	0.0250 (17)	−0.0039 (16)	0.0022 (14)	0.0053 (15)
C9	0.029 (2)	0.0337 (19)	0.0345 (19)	0.0021 (15)	0.0055 (15)	−0.0005 (16)
C10	0.034 (2)	0.0300 (18)	0.0306 (19)	−0.0040 (16)	0.0103 (15)	−0.0032 (16)
C11	0.039 (2)	0.0210 (16)	0.0211 (17)	−0.0035 (15)	0.0035 (14)	0.0017 (14)
C12	0.0273 (19)	0.0165 (15)	0.0277 (18)	−0.0018 (14)	0.0016 (14)	−0.0037 (14)
C13	0.036 (2)	0.0268 (18)	0.0195 (16)	0.0079 (15)	0.0070 (14)	0.0024 (14)
C14	0.034 (2)	0.0289 (18)	0.0265 (18)	0.0023 (15)	0.0046 (14)	0.0085 (15)
C15	0.0284 (19)	0.038 (2)	0.0251 (17)	0.0109 (16)	0.0115 (14)	0.0097 (16)
C16	0.035 (2)	0.0345 (19)	0.0229 (17)	0.0092 (16)	0.0076 (14)	0.0015 (15)
C17	0.0302 (19)	0.0282 (17)	0.0212 (16)	0.0005 (15)	0.0031 (13)	0.0029 (14)
C18	0.0244 (18)	0.0294 (18)	0.0202 (16)	0.0012 (15)	0.0046 (13)	0.0049 (14)
C19	0.034 (2)	0.0234 (17)	0.0234 (17)	0.0015 (14)	0.0048 (14)	−0.0048 (14)
C20	0.032 (2)	0.035 (2)	0.0316 (19)	−0.0015 (15)	0.0131 (15)	−0.0010 (16)
C21	0.024 (2)	0.043 (2)	0.038 (2)	−0.0020 (16)	0.0079 (15)	−0.0034 (17)
C22	0.034 (2)	0.0298 (19)	0.0292 (18)	0.0025 (15)	0.0028 (15)	−0.0009 (15)
C23	0.034 (2)	0.0200 (17)	0.0274 (17)	0.0011 (14)	0.0107 (14)	−0.0061 (14)
C24	0.0236 (19)	0.0209 (17)	0.0316 (18)	−0.0014 (14)	0.0090 (14)	−0.0056 (14)
C25	0.037 (2)	0.0294 (18)	0.0289 (18)	0.0020 (16)	0.0050 (15)	−0.0006 (15)
C26	0.039 (2)	0.037 (2)	0.031 (2)	−0.0001 (17)	0.0063 (16)	−0.0057 (18)
C27	0.036 (3)	0.080 (3)	0.068 (3)	−0.004 (2)	0.014 (2)	−0.002 (3)
C28	0.057 (3)	0.047 (2)	0.047 (2)	0.013 (2)	−0.004 (2)	−0.013 (2)
C29	0.085 (4)	0.065 (3)	0.079 (4)	0.032 (3)	−0.005 (3)	−0.006 (3)
C30	0.120 (4)	0.050 (3)	0.099 (4)	0.039 (3)	0.028 (3)	0.011 (3)
C31	0.030 (2)	0.052 (2)	0.036 (2)	0.0029 (17)	0.0106 (15)	−0.0085 (18)
C32	0.031 (2)	0.037 (2)	0.038 (2)	0.0011 (16)	0.0076 (16)	−0.0040 (17)
C33	0.045 (3)	0.067 (3)	0.057 (3)	0.006 (2)	−0.005 (2)	−0.001 (2)
C34	0.034 (2)	0.0312 (19)	0.048 (2)	−0.0075 (16)	0.0108 (16)	−0.0112 (17)
C35	0.037 (2)	0.046 (2)	0.037 (2)	−0.0095 (17)	0.0083 (16)	−0.0046 (18)
C36	0.046 (3)	0.057 (3)	0.050 (2)	−0.017 (2)	0.0107 (19)	−0.012 (2)
O1	0.0318 (13)	0.0296 (12)	0.0246 (12)	0.0010 (10)	0.0075 (9)	0.0009 (10)
O2	0.0325 (14)	0.0259 (12)	0.0321 (13)	0.0025 (10)	0.0067 (10)	0.0012 (10)
O3	0.0303 (14)	0.0371 (13)	0.0271 (12)	0.0034 (10)	0.0117 (10)	0.0021 (11)
O4	0.0267 (13)	0.0298 (12)	0.0350 (13)	−0.0019 (10)	0.0077 (10)	−0.0067 (10)
O5	0.0452 (17)	0.0675 (19)	0.0517 (17)	−0.0077 (14)	0.0100 (13)	0.0237 (15)
O6	0.0332 (16)	0.0567 (17)	0.0619 (18)	0.0023 (13)	0.0050 (13)	0.0099 (14)
O7	0.0580 (19)	0.086 (2)	0.0473 (17)	0.0177 (16)	0.0002 (14)	−0.0265 (17)
O8	0.0379 (16)	0.0612 (17)	0.0436 (15)	0.0107 (13)	0.0006 (12)	−0.0088 (14)
S1	0.0435 (5)	0.0230 (4)	0.0264 (4)	0.0019 (4)	0.0135 (4)	0.0013 (4)
S2	0.0447 (6)	0.0226 (4)	0.0313 (5)	−0.0038 (4)	0.0131 (4)	−0.0007 (4)
S3	0.0530 (6)	0.0247 (4)	0.0237 (4)	0.0024 (4)	0.0095 (4)	0.0026 (4)
S4	0.0393 (5)	0.0310 (5)	0.0258 (4)	−0.0033 (4)	0.0023 (4)	−0.0023 (4)

### Geometric parameters (Å, º)

**Table:**

Br1—C3	1.902 (3)	C22—H22	0.9500
Br2—C15	1.900 (3)	C23—C24	1.396 (4)
C1—C2	1.389 (4)	C23—S2	1.787 (3)
C1—C6	1.404 (4)	C24—O4	1.368 (3)
C1—S2	1.776 (3)	C25—O1	1.411 (3)
C2—C3	1.381 (4)	C25—C26	1.501 (5)
C2—H2	0.9500	C25—H25A	0.9900
C3—C4	1.375 (4)	C25—H25B	0.9900
C4—C5	1.398 (4)	C26—O5	1.189 (4)
C4—H4	0.9500	C26—O6	1.329 (4)
C5—C6	1.394 (4)	C27—O6	1.440 (4)
C5—S1	1.776 (3)	C27—H27A	0.9800
C6—O1	1.367 (3)	C27—H27B	0.9800
C7—C8	1.385 (4)	C27—H27C	0.9800
C7—C12	1.399 (4)	C28—C29	1.442 (5)
C7—S1	1.779 (3)	C28—O2	1.447 (4)
C8—C9	1.374 (4)	C28—H28A	0.9900
C8—H8	0.9500	C28—H28B	0.9900
C9—C10	1.391 (4)	C29—C30	1.505 (5)
C9—H9	0.9500	C29—H29A	0.9900
C10—C11	1.383 (4)	C29—H29B	0.9900
C10—H10	0.9500	C30—H30A	0.9800
C11—C12	1.394 (4)	C30—H30B	0.9800
C11—S3	1.785 (3)	C30—H30C	0.9800
C12—O2	1.373 (3)	C31—O3	1.432 (4)
C13—C14	1.392 (4)	C31—C32	1.494 (4)
C13—C18	1.395 (4)	C31—H31A	0.9900
C13—S3	1.782 (3)	C31—H31B	0.9900
C14—C15	1.377 (4)	C32—O7	1.188 (4)
C14—H14	0.9500	C32—O8	1.325 (4)
C15—C16	1.389 (4)	C33—O8	1.460 (4)
C16—C17	1.381 (4)	C33—H33A	0.9800
C16—H16	0.9500	C33—H33B	0.9800
C17—C18	1.400 (4)	C33—H33C	0.9800
C17—S4	1.783 (3)	C34—O4	1.437 (3)
C18—O3	1.374 (4)	C34—C35	1.496 (4)
C19—C24	1.393 (4)	C34—H34A	0.9900
C19—C20	1.393 (4)	C34—H34B	0.9900
C19—S4	1.791 (3)	C35—C36	1.526 (4)
C20—C21	1.379 (4)	C35—H35A	0.9900
C20—H20	0.9500	C35—H35B	0.9900
C21—C22	1.373 (4)	C36—H36A	0.9800
C21—H21	0.9500	C36—H36B	0.9800
C22—C23	1.388 (4)	C36—H36C	0.9800
			
C2—C1—C6	119.2 (3)	C26—C25—H25A	108.9
C2—C1—S2	119.4 (2)	O1—C25—H25B	108.9
C6—C1—S2	121.4 (2)	C26—C25—H25B	108.9
C3—C2—C1	120.2 (3)	H25A—C25—H25B	107.7
C3—C2—H2	119.9	O5—C26—O6	124.6 (3)
C1—C2—H2	119.9	O5—C26—C25	126.1 (3)
C4—C3—C2	120.9 (3)	O6—C26—C25	109.3 (3)
C4—C3—Br1	118.3 (2)	O6—C27—H27A	109.5
C2—C3—Br1	120.7 (2)	O6—C27—H27B	109.5
C3—C4—C5	119.9 (3)	H27A—C27—H27B	109.5
C3—C4—H4	120.1	O6—C27—H27C	109.5
C5—C4—H4	120.1	H27A—C27—H27C	109.5
C6—C5—C4	119.5 (3)	H27B—C27—H27C	109.5
C6—C5—S1	121.6 (2)	C29—C28—O2	111.4 (3)
C4—C5—S1	118.8 (2)	C29—C28—H28A	109.3
O1—C6—C5	122.9 (3)	O2—C28—H28A	109.3
O1—C6—C1	116.7 (3)	C29—C28—H28B	109.3
C5—C6—C1	120.1 (3)	O2—C28—H28B	109.3
C8—C7—C12	119.5 (3)	H28A—C28—H28B	108.0
C8—C7—S1	121.9 (2)	C28—C29—C30	113.1 (4)
C12—C7—S1	118.5 (2)	C28—C29—H29A	108.9
C9—C8—C7	120.9 (3)	C30—C29—H29A	108.9
C9—C8—H8	119.6	C28—C29—H29B	108.9
C7—C8—H8	119.6	C30—C29—H29B	108.9
C8—C9—C10	120.3 (3)	H29A—C29—H29B	107.8
C8—C9—H9	119.9	C29—C30—H30A	109.5
C10—C9—H9	119.9	C29—C30—H30B	109.5
C11—C10—C9	119.3 (3)	H30A—C30—H30B	109.5
C11—C10—H10	120.3	C29—C30—H30C	109.5
C9—C10—H10	120.3	H30A—C30—H30C	109.5
C10—C11—C12	120.8 (3)	H30B—C30—H30C	109.5
C10—C11—S3	118.9 (2)	O3—C31—C32	113.9 (3)
C12—C11—S3	120.2 (2)	O3—C31—H31A	108.8
O2—C12—C11	121.4 (3)	C32—C31—H31A	108.8
O2—C12—C7	119.4 (3)	O3—C31—H31B	108.8
C11—C12—C7	119.2 (3)	C32—C31—H31B	108.8
C14—C13—C18	119.8 (3)	H31A—C31—H31B	107.7
C14—C13—S3	118.3 (2)	O7—C32—O8	124.6 (3)
C18—C13—S3	121.8 (2)	O7—C32—C31	126.5 (3)
C15—C14—C13	119.6 (3)	O8—C32—C31	108.9 (3)
C15—C14—H14	120.2	O8—C33—H33A	109.5
C13—C14—H14	120.2	O8—C33—H33B	109.5
C14—C15—C16	121.1 (3)	H33A—C33—H33B	109.5
C14—C15—Br2	118.3 (2)	O8—C33—H33C	109.5
C16—C15—Br2	120.6 (2)	H33A—C33—H33C	109.5
C17—C16—C15	119.2 (3)	H33B—C33—H33C	109.5
C17—C16—H16	120.4	O4—C34—C35	107.3 (3)
C15—C16—H16	120.4	O4—C34—H34A	110.3
C16—C17—C18	120.4 (3)	C35—C34—H34A	110.3
C16—C17—S4	120.1 (2)	O4—C34—H34B	110.3
C18—C17—S4	119.5 (2)	C35—C34—H34B	110.3
O3—C18—C13	122.4 (3)	H34A—C34—H34B	108.5
O3—C18—C17	118.2 (3)	C34—C35—C36	111.7 (3)
C13—C18—C17	119.2 (3)	C34—C35—H35A	109.3
C24—C19—C20	119.9 (3)	C36—C35—H35A	109.3
C24—C19—S4	119.1 (2)	C34—C35—H35B	109.3
C20—C19—S4	121.0 (3)	C36—C35—H35B	109.3
C21—C20—C19	120.0 (3)	H35A—C35—H35B	107.9
C21—C20—H20	120.0	C35—C36—H36A	109.5
C19—C20—H20	120.0	C35—C36—H36B	109.5
C22—C21—C20	120.4 (3)	H36A—C36—H36B	109.5
C22—C21—H21	119.8	C35—C36—H36C	109.5
C20—C21—H21	119.8	H36A—C36—H36C	109.5
C21—C22—C23	120.6 (3)	H36B—C36—H36C	109.5
C21—C22—H22	119.7	C6—O1—C25	121.1 (2)
C23—C22—H22	119.7	C12—O2—C28	114.1 (2)
C22—C23—C24	119.5 (3)	C18—O3—C31	116.6 (2)
C22—C23—S2	119.5 (2)	C24—O4—C34	115.6 (2)
C24—C23—S2	121.0 (2)	C26—O6—C27	115.2 (3)
O4—C24—C19	119.9 (3)	C32—O8—C33	117.4 (3)
O4—C24—C23	120.2 (3)	C5—S1—C7	98.88 (14)
C19—C24—C23	119.6 (3)	C1—S2—C23	101.78 (14)
O1—C25—C26	113.4 (3)	C13—S3—C11	97.62 (14)
O1—C25—H25A	108.9	C17—S4—C19	99.14 (14)
			
C6—C1—C2—C3	−0.3 (4)	C20—C21—C22—C23	−0.2 (5)
S2—C1—C2—C3	177.8 (2)	C21—C22—C23—C24	0.0 (5)
C1—C2—C3—C4	3.6 (5)	C21—C22—C23—S2	−179.0 (2)
C1—C2—C3—Br1	178.8 (2)	C20—C19—C24—O4	−176.6 (3)
C2—C3—C4—C5	−2.4 (5)	S4—C19—C24—O4	3.6 (4)
Br1—C3—C4—C5	−177.8 (2)	C20—C19—C24—C23	−1.6 (4)
C3—C4—C5—C6	−1.9 (5)	S4—C19—C24—C23	178.6 (2)
C3—C4—C5—S1	174.3 (2)	C22—C23—C24—O4	175.9 (3)
C4—C5—C6—O1	178.0 (3)	S2—C23—C24—O4	−5.2 (4)
S1—C5—C6—O1	1.8 (4)	C22—C23—C24—C19	0.9 (4)
C4—C5—C6—C1	5.2 (4)	S2—C23—C24—C19	179.9 (2)
S1—C5—C6—C1	−170.9 (2)	O1—C25—C26—O5	−13.4 (5)
C2—C1—C6—O1	−177.3 (3)	O1—C25—C26—O6	167.7 (2)
S2—C1—C6—O1	4.7 (4)	O2—C28—C29—C30	171.2 (4)
C2—C1—C6—C5	−4.1 (4)	O3—C31—C32—O7	12.0 (5)
S2—C1—C6—C5	177.9 (2)	O3—C31—C32—O8	−169.7 (3)
C12—C7—C8—C9	0.7 (4)	O4—C34—C35—C36	−178.0 (3)
S1—C7—C8—C9	−177.3 (2)	C5—C6—O1—C25	63.4 (4)
C7—C8—C9—C10	0.4 (5)	C1—C6—O1—C25	−123.6 (3)
C8—C9—C10—C11	−0.5 (5)	C26—C25—O1—C6	54.1 (4)
C9—C10—C11—C12	−0.6 (5)	C11—C12—O2—C28	80.2 (4)
C9—C10—C11—S3	176.8 (2)	C7—C12—O2—C28	−103.3 (3)
C10—C11—C12—O2	178.3 (3)	C29—C28—O2—C12	−160.9 (3)
S3—C11—C12—O2	0.9 (4)	C13—C18—O3—C31	−64.3 (4)
C10—C11—C12—C7	1.7 (4)	C17—C18—O3—C31	121.5 (3)
S3—C11—C12—C7	−175.7 (2)	C32—C31—O3—C18	−66.1 (4)
C8—C7—C12—O2	−178.3 (3)	C19—C24—O4—C34	−86.5 (3)
S1—C7—C12—O2	−0.3 (4)	C23—C24—O4—C34	98.5 (3)
C8—C7—C12—C11	−1.7 (4)	C35—C34—O4—C24	169.0 (3)
S1—C7—C12—C11	176.3 (2)	O5—C26—O6—C27	−4.5 (5)
C18—C13—C14—C15	1.1 (4)	C25—C26—O6—C27	174.4 (3)
S3—C13—C14—C15	−175.3 (2)	O7—C32—O8—C33	0.9 (5)
C13—C14—C15—C16	5.0 (5)	C31—C32—O8—C33	−177.5 (3)
C13—C14—C15—Br2	−177.2 (2)	C6—C5—S1—C7	58.3 (3)
C14—C15—C16—C17	−4.0 (5)	C4—C5—S1—C7	−117.9 (3)
Br2—C15—C16—C17	178.2 (2)	C8—C7—S1—C5	33.4 (3)
C15—C16—C17—C18	−3.0 (4)	C12—C7—S1—C5	−144.6 (2)
C15—C16—C17—S4	175.4 (2)	C2—C1—S2—C23	131.6 (3)
C14—C13—C18—O3	178.0 (3)	C6—C1—S2—C23	−50.4 (3)
S3—C13—C18—O3	−5.8 (4)	C22—C23—S2—C1	−72.7 (3)
C14—C13—C18—C17	−7.9 (4)	C24—C23—S2—C1	108.3 (3)
S3—C13—C18—C17	168.4 (2)	C14—C13—S3—C11	118.2 (3)
C16—C17—C18—O3	−176.7 (3)	C18—C13—S3—C11	−58.1 (3)
S4—C17—C18—O3	4.9 (4)	C10—C11—S3—C13	−56.9 (3)
C16—C17—C18—C13	8.9 (4)	C12—C11—S3—C13	120.5 (3)
S4—C17—C18—C13	−169.5 (2)	C16—C17—S4—C19	−101.1 (3)
C24—C19—C20—C21	1.3 (5)	C18—C17—S4—C19	77.2 (3)
S4—C19—C20—C21	−178.8 (2)	C24—C19—S4—C17	−137.2 (3)
C19—C20—C21—C22	−0.4 (5)	C20—C19—S4—C17	43.0 (3)

### Hydrogen-bond geometry (Å, º)

**Table:**

*D*—H···*A*	*D*—H	H···*A*	*D*···*A*	*D*—H···*A*
C4—H4···S3^i^	0.95	2.91	3.755 (3)	149
C33—H33*A*···O7^ii^	0.98	2.58	3.551 (5)	169

Symmetry codes: (i) *x*, −*y*+1/2, *z*−1/2; (ii) −*x*+1, −*y*+1, −*z*+2.
